# Primary Antibody Deficiencies With Pulmonary Complications: A Single-Center Experience

**DOI:** 10.7759/cureus.21140

**Published:** 2022-01-12

**Authors:** Güzin Özden, Pelin Pınar Deniz, Pelin Duru Cetinkaya

**Affiliations:** 1 Allergy and Immunology, Adana City Training and Research Hospital, Adana, TUR; 2 Department of Pulmonology, Çukurova University Faculty of Medicine, Adana, TUR

**Keywords:** prımary antibody deficiency, interstitial lung, computed tomography, bronchiectasis, primary immunodeficiency

## Abstract

Background

Primary immunodeficiencies are a heterogeneous group of genetic diseases caused by one or more abnormalities in the immune system. Although pulmonary complications are common in patients with primary immunodeficiency diseases, these complications contribute significantly to morbidity and mortality.

Aim

The aim of our study was to evaluate the distribution of the features of pulmonary radiological involvement and demographic findings in this patient group.

Materials and methods

The files of patients who were treated and followed up with the diagnosis of primary immunodeficiency between 2014 and 2021 were analyzed retrospectively. Demographic data, symptoms, additional diseases, and computed tomography findings of the patients were recorded.

Results

The mean age of 32 cases was 37.34±13.54 (20-69) and the age of diagnosis was 28.90±15.75 (1-62). Twenty of the cases were male and 10 were female. The most common symptom was diarrhea with 53.1% and cough with 34.4%. The most common radiological finding is bronchiectasis in 75% of cases. Twenty-one (65.6%) of the cases had recurrent pneumonia before diagnosis and no pneumonia was observed after intravenous ımmunoglobulin replacement therapy. Three of the cases (9.4%) died during the follow-up.

Conclusions

Primary immunodeficiency should be considered in patients with bronchiectasis and a history of recurrent pneumonia, and further investigations should be performed. Early diagnosis of patients with primary immunodeficiency is very important for the early detection and treatment of malignancy and the interstitial lung disease that may develop.

## Introduction

Primary immunodeficiency diseases (PIDs) are rare, and more than 330 heterogeneous diseases with 320 different genetic defects have been described [1]. Although the main clinical findings are recurrent and chronic infections, the incidence of autoimmune, lymphoproliferative disorders, and malignancies has also increased in patients. Although pulmonary complications are common in patients with primary immunodeficiency diseases, these complications contribute significantly to morbidity and mortality. Common variable immunodeficiency (CVID) is the most common primary antibody deficiencies (PADs) subgroup with severe and recurrent sinopulmonary infections, poor vaccination response, low total serum immunoglobulin G (IgG) and IgA, and/or IgM levels [2]. This study examines the rate of pulmonary infections and their radiological findings in PADs. The aim of our study was to evaluate the pulmonary radiological features, comorbid symptoms, and diseases with the demographic characteristics of this patient group.

## Materials and methods

After the approval of the ethics committee, the files of patients who were treated and followed up by us with the diagnosis of primary immunodeficiency between 2014 and 2021 were retrospectively analyzed. Demographic data, symptoms, additional diseases, and computed tomography findings of the patients were recorded.

Recurrent pneumonia: patients diagnosed with two or more episodes of pneumonia in one year were termed recurrent pneumonia. Demographic data and radiological findings of groups with and without bronchiectasis were compared.

Statistics

Statistical data analysis was performed using the SPSS 20.0 (Statistical Package for the Social Sciences; IBM Corp., Armonk, NY) software. Only descriptive statistics were used in the analysis of the data. In evaluating the data, mean and standard deviation were used to describe continuous data while categorical variables were presented as frequencies and percentages.

## Results

The mean age of 32 cases was 37.34±13.54 (20-69) and the age of diagnosis was 28.90±15.75 (1-62). Twenty of the cases were male and 10 were female. Body mass index was 24.03±5.00 (16-36). The most common symptom was diarrhea (53.1%) and cough (34.4%). Thirty-seven point five percent (37.5%) of the cases have hepatomegaly and 53.1% of them have splenomegaly. The most common comorbidity is an obstructive pulmonary disease (asthma and chronic obstructive pulmonary disease (COPD)) with 21.87%. The most radiological finding was bronchiectasis in 75% of cases.

Demographic data are displayed in Table 1.

**Table 1:** Demographic characteristics of primary immunodeficiencies patients

Demographic features		N: 32 (100%)
Age (Year) (Mean ± SD, Min-Max)		37.34±13.54 (20-69 )
Mean age at diagnosis (Year) (Mean ± SD, Min-Max)		28.90±15.75 (1-62 )
Body mass index kg/m^2^(Min-Max)		24.03±5 (16-36 )
Male/Female		20(62.5%) /12(37.5%)
Recurrent pneumonia before diagnosis		21(65.6%)
Family history		6(18.8%)
Cough		11(34.4%)
Sputum		8(25%)
Dyspnea		6(18.8%)
Diarrhea		17(53.1%)
Weight loss		4(2.5%)
Exitus		3(9.4%)
Coronavirus disease 2019 disease history		8(25%)
Hepatomegaly		12(37.5%)
Splenomegaly		17(53.1%)
Comorbidity	Obstructive lung disease	7 (21.87%)
	Rheumatological disease	1 (3.1%)
	Autoimmune diseases	2 (6,25%)
	Malignant (lymphoma, maltoma)	2 (6.25%)
	Chronic hepatitis	1 (3.1%)
	Celiac disease	3 (9.37%)
	Chron disease	1 (3.1%)
	Heart disease	2 (6,25%)
	Sarcoidosis	1 (3.1%)
	Epilepsy	1 (3.1%)
	Interstitial lung disease	1 (3.1%)

**Figure 1:**
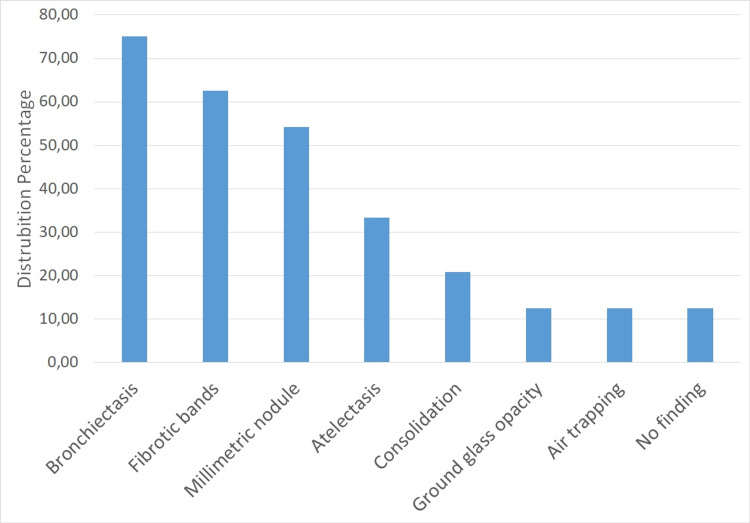
Distribution of radiological findings

When the thorax computed tomography findings of the patients were examined, bronchiectasis in 75%, fibrotic band in 62.5%, millimetric nodule in 54.16%, atelectasis in 33.33%, consolidation in 20%, ground glass in 12.50%, and 12.5% air trapping was observed. No pathological tomography findings were detected in 12.5% of the cases (Figure 1).

Twenty-one (65.6%) of the cases had recurrent pneumonia before diagnosis and no pneumonia was observed after intravenous immunoglobulin (IVIG) treatment. Twenty-five percent (25%) of the cases had coronavirus disease 2019 disease (COVID-19) and were asymptomatic. No deaths due to COVID-19 were observed.

Since the most radiological finding was bronchiectasis, patients were divided into two groups - those with and without bronchiectasis, and no statistically significant difference was found between the groups when age, gender, BMI, presence of recurrent pneumonia before diagnosis, hepatomegaly, or splenomegaly were compared (p≥0.005) (Table 2).

**Table 2:** Comparison of patients' variables according to bronchiectasis status

	Bronchiectasis N:24	No bronchiectasis N:8
Age (Year) (Mean ± SD, Min-Max)	37.7±15.0 (20-69)	36.25±8.39 (24-44)
Mean age at diagnosis (Year) (Mean ± SD, Min-Max)	28.17±17.01 (1-62)	31.62±11.69 (8-42)
Body Mass Index- kg/m2 (Mean ± SD, Min-Max)	23.70±5.47 (15-36)	25±3.33 (18-29)
Male/ Female	62.5%/ 37.5%	62.5% /37.5%
Recurrent pneumonia before diagnosis	70.8 %	50.0%
Family history	20.83%	12.5%
Cough	33.3%	37.5%
Sputum	25%	25%
Dyspnea	16.76 %	25%
Diarrhea	58.3%	37.5%
Weight loss	16.7%	0%
Exitus	8.3%	12.5%
Coronavirus disease 2019 disease history	25%	25%
Hepatomegaly	45.8%	75%
Splenomegaly	33.3%	50 %

When the comorbidity of the cases was examined, 21.87% had obstructive pulmonary disease with an autoimmune disease in 6.25%, malignancy in 6.25%, and celiac disease in 9.37%. Three of the cases (9.4%) died during the follow-up. Two patients died from malignancy while one patient died from cytomegalovirus-related gastroenteritis.

## Discussion

Primary immunodeficiency diseases include immune defects, most of which are hereditary [3]. Predominantly, antibody deficiencies are the most common primary immunodeficiencies, which are caused by genetic disorders and result in an impaired immune system [4]. In this study, PIDs with antibody deficiencies were examined.

Epidemiological studies of PIDs are rare, especially in adults. The frequency of PIDs in the European Union is estimated to be 1/10,000 people [5]. However, experts suggest that the real frequency of those diseases is much higher [6]. The prevalence of PIDs in the general population in Turkey is not clearly known. Kilic et al. reported an estimated cumulative prevalence rate of 30.5/100,000 for all forms of PID in the general population. In this study, 1275 patients were younger than 18 years, 85 of them were older than 18 years, and the most common subgroup was found to be antibody deficiency (22.7/100.000) [7].

The prognosis is better with the diagnosis and treatment of PIDs in childhood. However, it is assumed that 25%-40% of all PIDs are diagnosed in adults [6]. In this study, the mean age of diagnosis of PID was found to be 28.90 years. The object of this study is to draw attention to PID since PIDs are diagnosed late and epidemiological studies are scarce in adults in our country, hence raising awareness for early diagnosis.

Pulmonary complications are common in patients with PIDs and often contribute significantly to morbidity and mortality. Pulmonary infections are an important indication of PIDs. It has been reported that the most common initial presentation of PIDs is pneumonia. A history of two or more episodes of pneumonia in one year is the first warning sign of PIDs [8-9]. In this study, 21 cases (65.6%) had recurrent pneumonia before diagnosis. In addition to lung infections, patients with PID are at increased risk for other pulmonary complications, including asthma, bronchiectasis, interstitial lung disease, and malignancy. Especially, lymphoma and autoimmune diseases are common [10-12]. In our study, 21.87% of the patients had an obstructive pulmonary disease with an autoimmune disease in 6.25%, malignancy in 6.25%, and celiac disease in 9.37%. As survival from infection increases with early diagnosis and treatment, noninfectious pulmonary complications are more common in patients with PID. Obstructive airway disease, including asthma, bronchiolitis, and bronchiectasis, is quite common in PID. Interstitial lung disease (ILD) is also seen more frequently than in the normal population. In our cases, although obstructive pulmonary diseases are the most common comorbidities, the presence of malignancy, ILD, and autoimmune diseases are also observed. Three of our cases (9.4%) died during follow-up. The incidence of bronchiectasis in PID has been reported to be between 54% and 73% [13-15]. Consistent with the literature, bronchiectasis is the most common radiological finding in our patients (75%). The most common lung diseases in PID cases are bronchiectasis and atelectasis. Chest X-ray is insufficient to show the structural changes in the lung in PID cases, and high-resolution computed tomography should be used in cases where it is necessary to evaluate the lung parenchyma [16]. High-resolution computed tomography was also used in our study. On tomography, bronchiectasis was observed in 75%, fibrotic band in 62.5%, millimetric nodule in 54.16%, atelectasis in 33.33%, consolidation in 20%, ground glass in 12.50%, and air trapping in 12.5%. While the mean age at diagnosis was 28.17 in cases with bronchiectasis, it was 31.62 in the group without bronchiectasis. Although there was no statistically significant difference, the group with bronchiectasis was diagnosed earlier. This may be due to the fact that physicians have bronchiectasis and think that it may be PID and trigger it further.

IVIG therapy is used in the treatment of PIDs, especially in those with antibody deficiency. IVIG consists of immunoglobulins (mostly immunoglobulin G) purified from pooled human plasma [17]. The primary goal of immunoglobulin therapy in patients with primary immunodeficiency is the prevention of sepsis, pneumonia, and other serious acute bacterial infections [18]. Studies on immunoglobulin therapy have shown a decrease in the incidence of viral and bacterial upper respiratory tract infections and bronchitis, a decrease in antibiotic use, fewer hospitalizations, improvement in lung functions, and an increase in quality of life [19]. Regular treatment with immunoglobulin in patients with primary immunodeficiency is associated with higher health perception scores [20]. In our study, pneumonia was not observed in patients after IVIG treatment. Twenty-five percent (25%) of the cases had asymptomatic COVID-19. No deaths due to COVID-19 were observed.

IVIG therapy increases the life expectancy of patients, and less infectious complications are observed in primary immunodeficiency cases. Therefore, PID should be considered in the differential diagnosis in cases with recurrent pneumonia and radiological bronchiectasis.

The limitations of our study are that it is retrospective, with a small number of patients.

## Conclusions

Recurrent lung infections in undiagnosed primary immunodeficiency patients may lead to lung damage. Therefore primary immunodeficiency diseases should be considered in patients with bronchiectasis, interstitial lung disease, and a history of recurrent pneumonia, and further investigations should be performed. In our study, pneumonia was not detected with IVIG treatment in PID patients, and death due to COVID-19 did not occur. Early diagnosis is important not only for lung diseases but also for cancer, autoimmune diseases, and infections involving other organs. As a result, the awareness of professionals improves patients' quality of life.
